# HPV16 entry requires dynein for minus-end transport and utilizes kinesin Kif11 for plus-end transport along microtubules during mitosis

**DOI:** 10.1128/jvi.00937-24

**Published:** 2024-12-04

**Authors:** Timothy R. Keiffer, Stephen DiGiuseppe, Lucile Guion, Malgorzata Bienkowska-Haba, Katarzyna Zwolinska, Abida Siddiqa, Anand Kushwaha, Martin J. Sapp

**Affiliations:** 1 Department of Microbiology and Immunology, Center for Molecular and Tumor Virology, Feist-Weiller Cancer Center, Louisiana State University Health Sciences Center 547965 , Shreveport, Louisiana, USA; 2 Edward Via College of Osteopathic Medicine 689017 , Monroe, Louisiana, USA; University of Toronto, Toronto, Canada

**Keywords:** HPV16, microtubules, mitosis, dynein, kinesins, Kif11, Kif18a, Kif25, proximity ligation assay, knocksideways

## Abstract

Human papillomaviruses (HPVs) travel from the trans-Golgi network (TGN) to the condensed (mitotic) chromosomes during mitosis. Partially uncoated HPV capsids utilize a unique vesicular structure for trafficking and nuclear import, which is directed by the minor capsid protein L2. However, it is still unknown which precise factors facilitate post-TGN HPV trafficking to the nucleus. Herein, we analyzed HPV16-infected mitotic cells using high-resolution microscopy, coupled with motor protein inhibition, to further elaborate on post-TGN trafficking by tracking the location and/or quantification of EdU-labeled HPV pseudogenomes on microtubules, certain kinesins, and mitotic chromosomes. We also adapted a knocksideways approach to determine if L2 and Kif11 interact in infected cells. We visualized dynein co-localization with HPV pseudogenomes along mitotic microtubules and measured HPV pseudogenome accumulation after short-term dynein inhibition. Additional inhibitor studies implicated a specific kinesin, Kif11, as participating in HPV pseudogenome delivery to the nucleus. Short-term inhibition of Kif11 decreased HPV pseudogenome accumulation at mitotic chromatin. In addition, Kif11, along with kinesins Kif18a and Kif25, were in proximity to L2 during infection. While we were unable to determine a direct interaction between L2 and Kif11, we were able to show via knocksideways approach that relocalization of exogenous Kif11 decreased HPV pseudogenome accumulation to the mitotic chromatin. Our data support a model whereby HPV16 utilizes dynein for minus-end trafficking along mitotic microtubules and utilizes Kif11 for plus-end movement in the late stage of viral entry.

**IMPORTANCE:**

Human papillomaviruses (HPV) utilize a unique vesicular structure to shield their genomes from detection during trafficking from the trans-Golgi network (TGN) to the nucleus during mitosis. The exact cellular factors responsible for trafficking these HPV genome containing vesicles along mitotic microtubules via the L2 minor protein remain unknown. We show via high-resolution microscopy that pharmacological inhibition of dynein and the kinesin Kif11 significantly decreases HPV pseudogenome accumulation on mitotic chromatin. Several kinesins were detected in proximity to incoming HPV pseudogenomes. Finally, using a novel knocksideways approach, we show reduced HPV pseudogenome accumulation on mitotic chromatin upon Kif11 relocalization to the mitochondria. Herein, our data suggest HPV utilizes minus- and plus-end mediated trafficking along mitotic microtubules to complete its genome trafficking to the nucleus.

## INTRODUCTION

Human papillomaviruses (HPVs) are small, non-enveloped DNA viruses associated with hyperproliferative lesions of the skin and mucosa. While most lesions are benign or self-limiting, infection with high-risk HPV types can progress to anogenital or oropharyngeal squamous cell carcinomas (1, 2). During primary infection, HPV virions attach to heparan sulfate proteoglycans (HSPGs) on the extracellular matrix and/or cell surface (3–6). Multiple engagements with HSPGs result in conformational changes in both the major and minor capsid proteins, L1 and L2, respectively (7, 8). The N-terminus of L2 is exposed by host cell cyclophilin B, revealing a furin convertase cleavage site (9–11). The cleavage of these 12 amino acids is critical for downstream events in the entry process. Following various conformational changes and association with several non-HSPG secondary receptors, HPV virions are internalized via the endocytic route and then trafficked to the endosome (12–14). Low pH triggers partial virion uncoating, whereupon a subset of L1 protein remains associated with L2 protein and the viral genome, most likely as capsomeres (15–18). The L2 protein then undergoes more conformational changes, resulting in the translocation of a majority of its C-terminus through the endosomal membrane; this translocation is promoted by a membrane-destabilizing domain (residues 445–467) and a putative transmembrane domain (residues 45–65) in L2, as well as the newly described chaperone function of y-secretase (19–23). This translocation event allows the large C-terminal region (residues 66–473) of L2 to be cytoplasmic and interact with cytosolic factors, such as retromer complex to facilitate trafficking to the trans-Golgi network (TGN) (24–30). There is some evidence that HPV also traffics to the endoplasmic reticulum (ER) (21, 31) after trafficking to the TGN (32). HPV does not utilize nuclear pore complexes to reach the nucleus, but rather mitosis-induced nuclear envelope breakdown, which was shown to be the key rate-limiting step (33, 34). Incoming viral genomes then take advantage of the reorganization of the cell to bud out of the TGN in a transport vesicle and associate with the condensed chromosomes (34, 35). After mitosis completion and nuclear envelope reformation, promyelocytic leukemia protein (PML) nuclear body proteins are recruited to HPV-harboring vesicles. Finally, viral genomes egress from the transport vesicle by an unknown mechanism allowing access to the viral DNA for subsequent transcription and replication (18, 35–38).

We hypothesized that HPV utilizes mitotic microtubules (MTs) to traffic from the TGN to the condensed chromosomes. MTs are dynamic cytoskeleton components involved in cell division and intracellular transport (39). MTs are polarized with their plus-ends acting as the growing ends at the cell periphery or attached to kinetochores during mitosis, while their minus-ends converge at the microtubule organizing center (MTOC) or centrosomes near the nucleus (40). Two classes of motor proteins mediate the directed transport of cargo on microtubules: dynein and kinesins. Dynein facilitates cargo transport towards the minus-ends, while kinesins transport cargo towards the plus-ends (41). We distinguish astral MTs from spindle MTs by their subcellular localization; astral MTs span from the cell periphery to the MTOC, and spindle MTs connect the MTOC to kinetochores (39). During mitosis, dynein is enriched on astral MTs, while mitotic kinesins are enriched on spindle MTs (40, 42). Other viruses known to utilize the MT network and motor proteins during infection are poliovirus (PV), influenza virus, herpes simplex virus 1 (HSV-1), and human immunodeficiency virus (HIV) (43). PV and influenza virus were shown to utilize dynein-mediated retrograde transport to the nuclear periphery (44, 45). HSV-1 was found to interact with dynein and kinesins as to traffic to the MTOC via minus-end-mediated transport, and then switch directionality and traffic towards the nucleus via plus-end-directed transport (46–48). In contrast, HIV utilizes kinesin-mediated retrograde transport along MTs (49). Our previous work and others have shown data of HPV-harboring transport vesicles associating with mitotic MTs and accumulating near the MTOC during mitosis (35, 50). More specifically, HPV genomes were observed localizing first along astral MTs during prophase, then followed by spindle MTs at later stages of mitosis. This suggested that HPV-harboring transport vesicles may be cargo for both motor proteins at different times during mitosis (35). Since most of the L2 protein is on the cytosolic side of transport vesicles and has been shown to interact with cellular factors to facilitate earlier tracking events, we hypothesized that the L2 protein interacts with dynein and kinesins during mitosis to mediate post-TGN trafficking events. In turn, this allows for MT-mediated trafficking of the HPV genome from the TGN to the MTOC and onward to condensed chromosomes, denoting a switch in directionality. To show further support for our hypothesis, a dynein-binding domain on L2, which has been previously identified at residues 456–461 on the C-terminus of the L2 protein, specifically interacts with dynein light chains DYNLT1 and DYNLT3 (51, 52). More recently, the Schelhaas group showed that knockdown of DYNLT3 in HeLa cells reduces HPV16 pseudovirus infectivity and subsequent L2 protein association with condensed chromosomes by 50%. They also demonstrated a novel complex that HPV utilizes for viral genome trafficking via dynein-mediated transport in an infection model (53). Several mitotic kinesins have also been identified via large-scale siRNA screens to be required for or affect HPV infection if they are knocked-down: Kif3a, Kif11, Kif18a, Kif23, and Kif25. However, no direct interactions of these kinesins with the L2 protein have been identified and their exact role(s) in HPV infection remain unknown (27, 34). Furthermore, a domain consisting of residues 188 to 334 on the L2 protein, aptly named “chromosome-binding region” (CBR), was shown to interact with mitotic chromosomes during mitosis (27, 50). Finally, we and others have characterized several point mutations within the nuclear retention signal (NRS) on L2 protein, consisting of residues 291–315 (28, 54). One identified mutant, referred to within as R302/5A, associated with astral MTs in prophase, but was specifically impaired in associating with spindle MTs in the late stages of mitosis (35).

Herein, we dissected HPV late trafficking events along MTs during mitosis. Using high-resolution microscopy and motor protein inhibitors, we provide evidence that HPV DNA associates with dynein on astral MTs during prophase, followed by kinesin-like protein Kif11 (also called Eg5 but referred to as Kif11 hereafter) on spindle MTs at later stages of mitosis. Furthermore, inhibiting dynein activity results in blocking accumulation of HPV pseudogenomes in proximity to the MTOC. Additionally, inhibiting Kif11 results in significantly less HPV pseudogenome accumulation on mitotic chromatin. In support of this, we also demonstrate that Kif11, along with other mitotic kinesins, are in proximity to the L2 protein during infection. Finally, we show that inducing relocalization of Kif11 via a recently described protocol called “knocksideways” significantly reduces HPV pseudogenome accumulation in the nucleus.

## RESULTS

### Incoming HPV pseudogenome co-localizes with mitotic microtubules in mitosis

**Fig 1:**
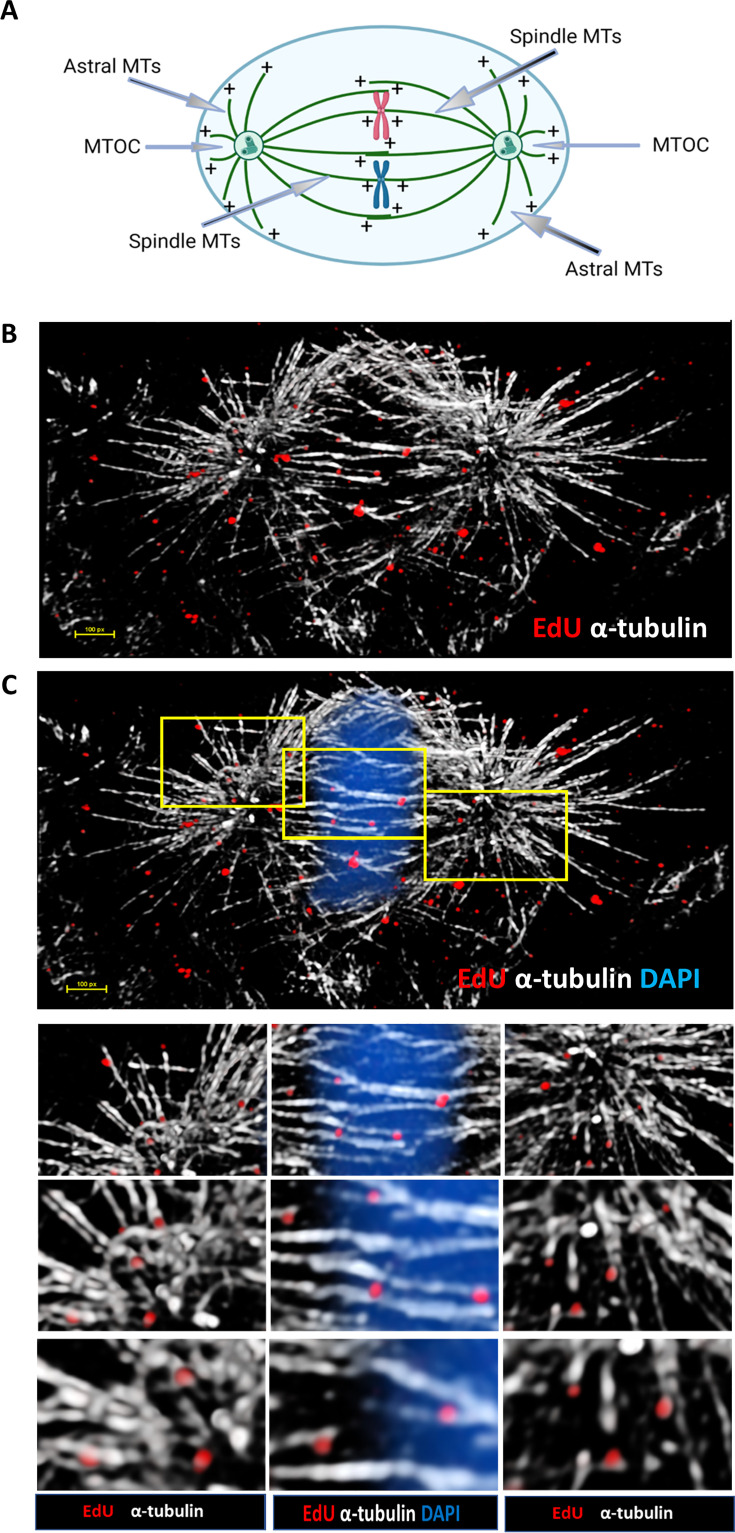
HPV colocalizes with mitotic microtubules during mitosis. (**A**) Simplified diagram gram showing relative locations of astral mitotic microtubules (MTs) and spindle MTs of a dividing eukaryotic cell during metaphase; astral MTs span from the microtubule organizing center(s) (MTOC) to the cell periphery, while spindle MTs span from the MTOCs to the kinetochores of the chromosomes situated in the middle of the diagram. The growing (+) ends of the MTs extending from the microtubule organizing center(s) (MTOC) are also marked. Diagram was generated using BioRender and its available templates. (**B**) HaCaT cells were infected with EdU-labeled PsVs for 24 h, fixed, permeabilized, and treated with Click-iT reaction buffer and AF555 dye to stain EdU-labeled pseudogenomes (red) as previously described in Materials and Methods. Cells were then stained using anti-α-tubulin antibody (white) and mounted in DAPI (not shown); an infected representative cell undergoing metaphase is depicted here. High resolution images were acquired using a Nikon N-SIM E Super Resolution microscope, 100× objective, and then processed with IMARIS for visualization. (**C**) Representative image from (**B**) with DAPI staining (blue) included. Three locations on this staining, as indicated with yellow boxes, are present to highlight the MTOCs with the astral MTs (left and right boxes) and the condensed chromosomes and the spindle MTs (middle box). Three representative close-ups per box are shown underneath the main staining to note presence of EdU-labeled genomes on astral (left and right inserts) and spindle (middle insert) microtubules, along with their proximity to the condensed chromosomes (DAPI).

In our previous work, we had observed HPV pseudogenomes aligning along astral and spindle MTs during prophase and metaphase using confocal microscopy (35). We revisited this observation using structured illumination microscopy (SIM) super-resolution fluorescence microscopy. HaCaT cells, a human immortalized keratinocyte cell line, were infected with EdU-labeled HPV16 pseudovirions (PsV16s) for 24 h, with the EdU stained for using Click-iT reaction chemistry, and α-tubulin was detected by immunofluorescence. Z-stacks of mitotic cells were acquired using a Nikon N-SIM-E high-resolution microscope. Next, we used the microscopy image analysis software IMARIS to visualize the MTs and EdU puncta in the three-dimensional space. A simple diagram showing astral MTs and spindle MTs during mitosis (metaphase) are displayed in Fig. 1A. These analyses confirmed a partial co-localization of viral pseudogenome with astral and spindle MTs at the prophase and metaphase stages of mitosis (Fig. 1B and C).

### Incoming HPV pseudogenome co-localizes with dynein in the early stages of mitosis

**Fig 2:**
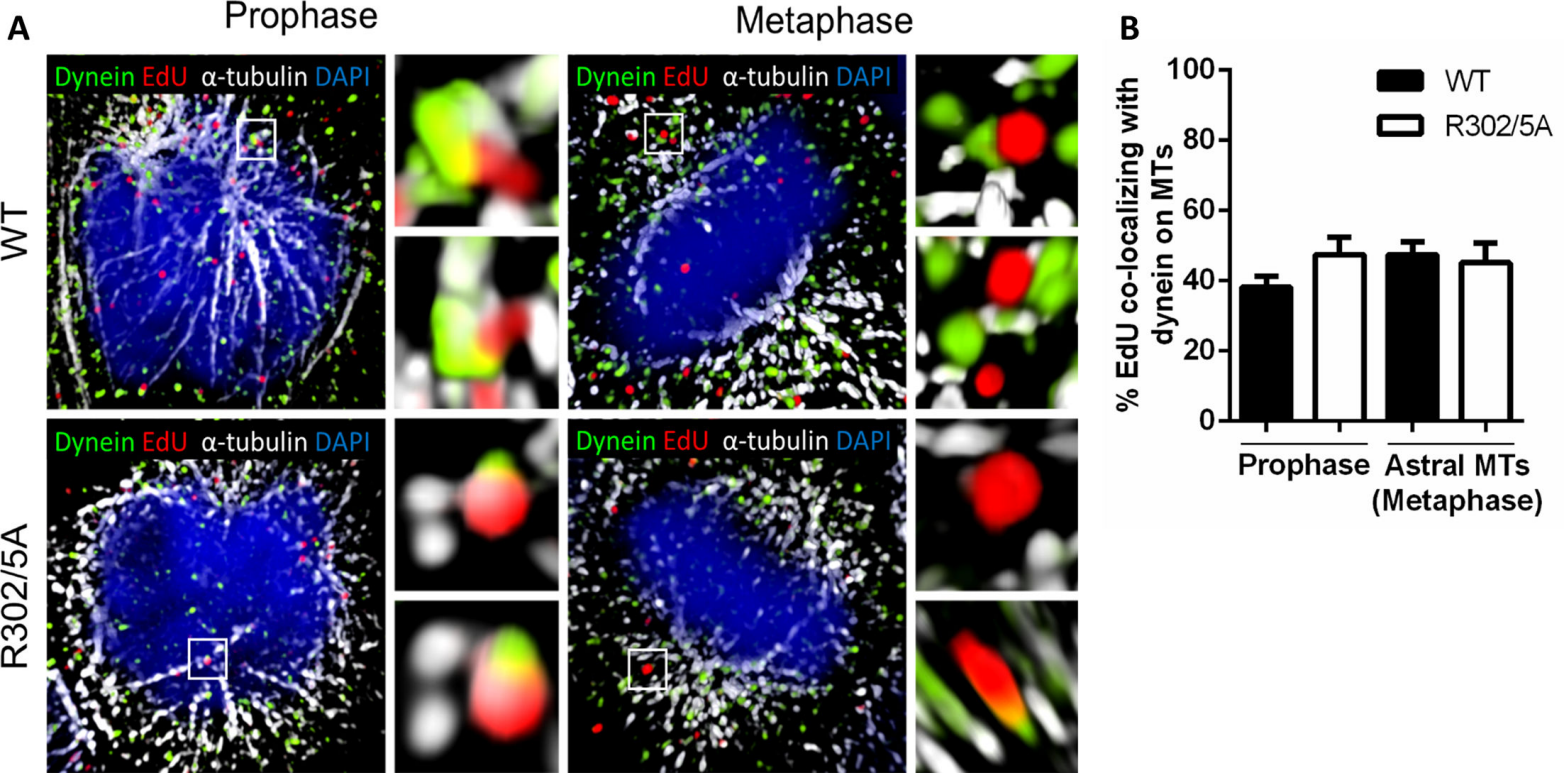
Pseudogenomes co-localize with dynein on mitotic microtubules during prophase and metaphase. HaCaT cells were infected with WT or R302/5A EdU-labeled PsVs for 24 h and subjected to the staining protocol along with the Click-iT reaction as previously described in Materials and Methods. AF555 dye was used to stain EdU-labeled pseudogenomes in cells (red), rabbit anti-dynein intermediate chain 1 antibody was used to stain for dynein (green), and mouse anti-α-tubulin antibody was used to stain for α-tubulin (white); cell nuclei were visualized using DAPI (blue). (**A**) Images show 3D reconstruction of high-resolution z-stacks, and close-up images were rotated at a 45° angle on the x-, y-, and z-axes in the 3D-rendered images. (**B**) Percent EdU co-localizing with dynein on MTs was determined with IMARIS by measuring the number of EdU puncta co-localizing with dynein foci on MTs over the total number of EdU puncta on MTs in prophase or astral MTs in metaphase. Prophase: WT = 38.23% ± 3.02%; R302/5A = 47.45% ± 4.98%. Metaphase: WT = 47.43% ± 3.69%; R302/5A = 45.18% ± 5.53%. Each quantification is shown as an average of two independent experiments and standard error to the mean (SEM), with 20–30 cells in each condition and experiment. *P*-value was determined using Student *t*-test. ns: *P* > 0.05.

Dynein is known to be enriched on astral MTs during mitosis (40, 42), and the L2 protein binds to dynein light chains (51–53). Given these findings, we sought to determine whether dynein facilitates trafficking of the HPV-harboring vesicle from the TGN to the MTOC, which would be observed in cells in the beginning of mitosis, e.g.*,* prophase. We acquired high-resolution images of HaCaT cells infected with HPV16 PsVs harboring an EdU-labeled pseudogenome after immunofluorescence staining. The resulting images are 3D reconstructed z-stacks depicting dynein (green), EdU-labeled viral pseudogenomes (red), and α-tubulin (white) (Fig. 2A). The cells were infected with either WT or R302/5A L2 mutant PsV16s. PsVs carrying this L2 mutation have been previously characterized and were shown to successfully traffic from the TGN to the MTOC, but they were not detected associating with the condensed chromosomes during mitosis (28, 35, 54). We quantified the percent of EdU puncta co-localizing with dynein along MTs compared with the total number of EdU puncta along MTs (Fig. 2B). We focused our analysis on cells in prophase, which corresponds to early mitotic trafficking of HPV-harboring vesicles toward the MTOC, and metaphase, which corresponds to later trafficking events after the switch in directionality from the MTOC to condensed chromosomes would have occurred. In metaphase cells, astral and spindle MTs are visually distinct, thus we focused our analysis on astral MTs only. We found that similar percentages, with no significant difference, of Edu-labeled genomes from both WT and R302/5A PsVs co-localized with dynein during prophase (38% and 47%, respectively) and metaphase (47% and 45%, respectively) (Fig. 2B), indicating the R302/5A mutation within the CBR is not affecting L2 binding to dynein.

**Fig 3:**
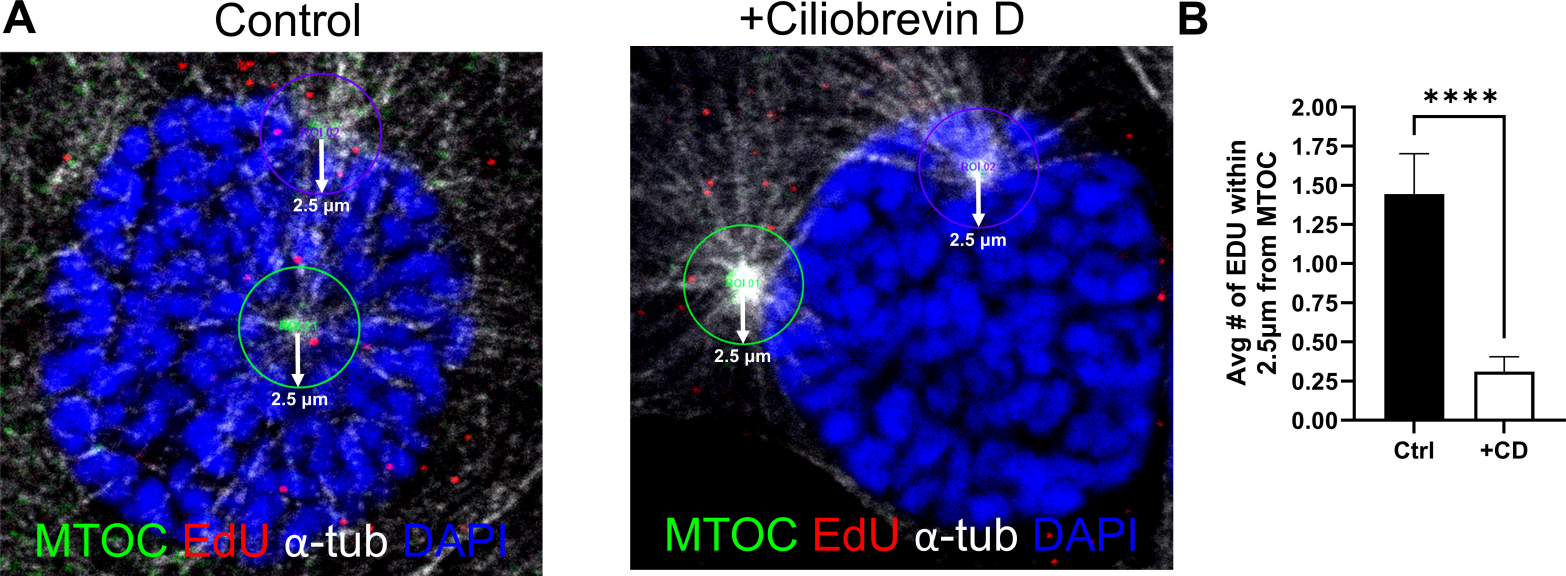
Inhibition of dynein motor protein blocks trafficking of pseudogenomes to the microtubule-organizing center. HaCaT cells were infected with WT EdU-labeled PsVs for 24 h. At 24hpi, the infected cells were treated with 50 µM ciliobrevin D, an inhibitor of dynein, for 10 min. Cells were then subjected to the staining and Click-iT protocol as previously described in Materials and Methods. EdU-labeled pseudogenomes were visualized with AF555 dye (red; EDU), while the MTOC was detected by staining using mouse AF488-conjugated anti-γ-tubulin antibody (green; MTOC); mouse anti-α-tubulin antibody was used to stain for α-tubulin (white; α-tub), and cell nuclei were visualized using DAPI (blue). (**A**) Confocal images were acquired for prophase cells only. In LAS AF, a region of interest (ROI) was drawn 2.5 µm radially away from each MTOC. (**B**) EdU puncta localization was determined by manually counting the number of EdU puncta located within each ROI and calculating the average. Results are shown as an average of three independent experiments and SEM with 10–20 ROIs counted per experiment. Ctrl = 1.44 ± .257; CD = 0.31 ± .094. *P*-values were determined using Student *t*-test. ****: *P* < 0.0001.

To further investigate the role of dynein in HPV trafficking, we infected HaCaT cells with EdU-labeled WT PsV16 for 24 h. At 24 h after infection, we treated the infected cells with an inhibitor of dynein motor function, ciliobrevin D (CD) (55), for 10 min (Fig. 3). We acquired confocal images showing the MTOC (green), EdU-labeled viral pseudogenomes (red), and α-tubulin (white) (Fig. 3A). We selected cells in prophase, and using LAS AF software, we measured the radial distances from the MTOC with a concentric circle corresponding to 2.5 µm from the MTOC. We quantified the number of EdU puncta present in each circle co-localizing with α-tubulin in control (Ctrl) and inhibitor-treated cells (Fig. 3B). We observed an average of 1.44 ± 0.257 EdU puncta at the MTOC compared with 0.311 ± 0.094 after dynein inhibitor treatment. This represented a 78.4% reduction in the average number of EdU puncta localizing at the MTOC after 10 min of CD treatment. These data suggest that dynein is co-opted by HPV to traffic the viral genome to the MTOC during prophase.

### Kinesin Kif11 is partially involved in plus-end-directed transport of HPV during mitosis

**Fig 4:**
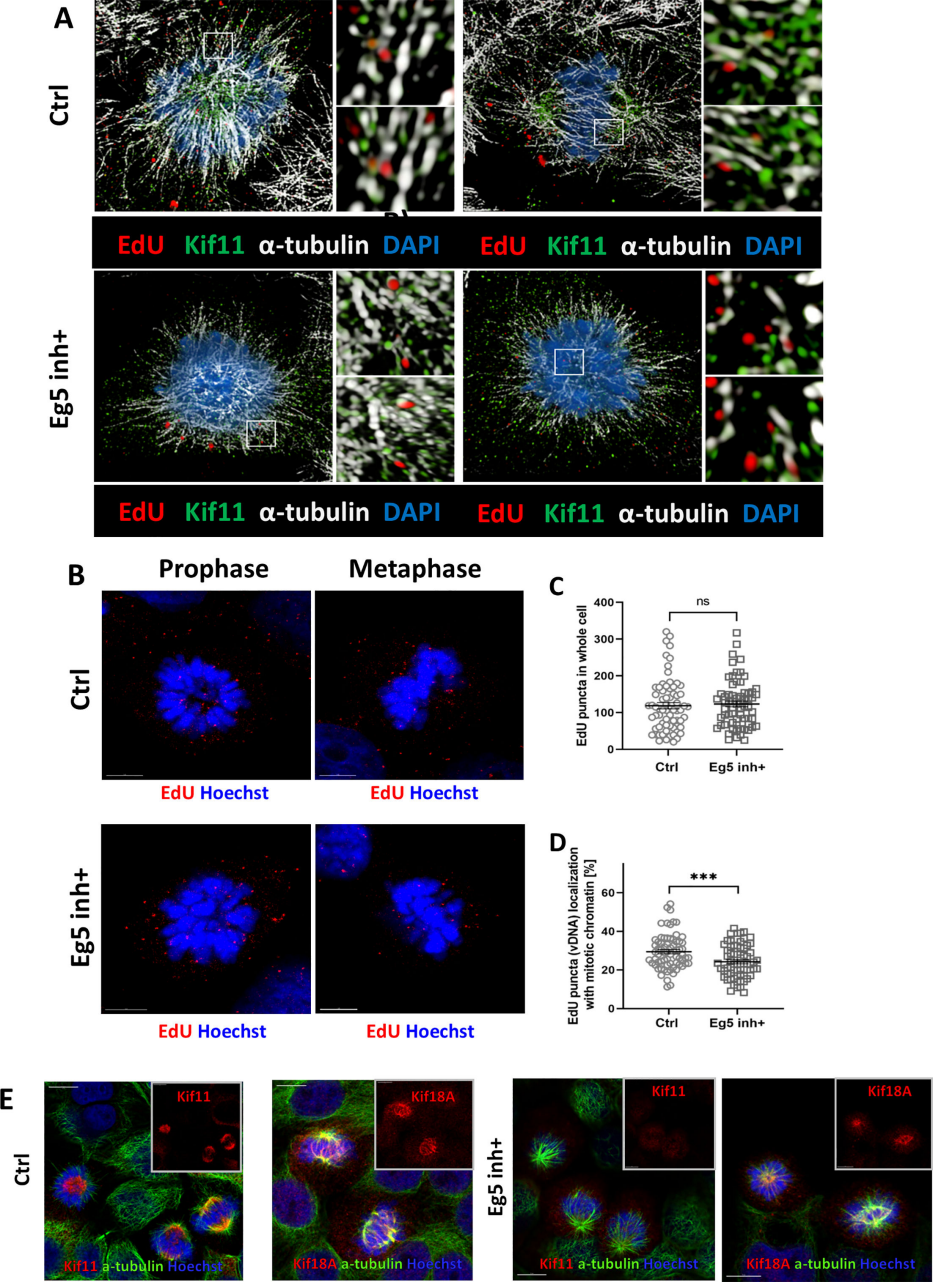
Kif11 inhibitor reduces transport of EdU-labeled PSVs to mitotic chromosomes. HaCaT cells were infected with WT EdU-labeled PsVs for 24 h and then treated with 1.5 µM Kif11 inhibitor (Eg5 inh+) or left untreated (Ctrl) for 10 min prior to the cells being stained and subjected to the Click-iT reaction as previously described in Materials and Methods. (**A**) AF555 dye was used to stain EdU-labeled pseudogenomes (red), mouse anti-α-tubulin antibody was used to stain for α-tubulin (white), and rabbit anti-Kif11 antibody was used to stain for Kif11 (green). Left-hand panel of Ctrl row of (**A**) is of an infected HaCaT cell in prophase, and the right-hand panel of Ctrl row of (**A**) is of an infected HaCaT cell in metaphase. Since Eg5 inh+ treatment locks cells into a monoastral phenotype (56), both the left-hand and right-panels of the Eg5 inh+ row of (**A**) are of pre-monoastral cells. Two smaller close-up images, rotated at a 45° angle on the x-, y-, and z-axes, are included on the right-side of the main image(s) to better show close association, or lack thereof in Eg5 inh+, between EdU-labeled HPV genome and Kif11. (**B**) AF555 dye was again used to stain EdU-labeled pseudogenomes in cells undergoing mitosis at prophase or metaphase (red), and mitotic chromosomes were visualized using Hoechst (blue). Images represent single medial slices. (**C**) Quantification of EdU-labeled HPV16 PsV in whole cells +/−Eg5 inh+ treatment; at least 20 cells per group, control and Eg5 inh+ were counted, with 69 total cells counted (group numbers of 21, 26, and 22). Localization of EdU puncta (red) was analyzed with IMARIS using spots/surface analysis. The number of pseudogenomes in whole cells was established based on alpha tubulin and EdU-labeled HPV genome signals; determining genome and the localization on mitotic chromosomes was based on Hoechst staining. Lines in graph represent mean with SEM, and statistical significance was assessed by Student *t*-test, *N* = 3, ns: *P* > 0.05. (**D**) Quantification of EdU-labeled HPV16 PsV (red) co-localized with mitotic chromatin (blue) upon Kif11 inhibitor treatment using IMARIS. At least 20 cells per group, control and Eg5 inh+ were counted, with 66 total cells counted (group numbers of 21, 25, and 20). Lines in graph represent mean with SEM, and statistical significance was assessed by Mann Whitney test, *N* = 3, ***: *P* < 0.001. (**E**) Uninfected HaCaT cells were treated with 1.5 µM Eg5 inhibitor for 10 min and then stained for Kif11 and Kif18a as previously described in Materials and Methods, except cells were also subjected to the Click-iT reaction, but without the AF555 dye to stain for kinesins under denaturing conditions. Either Kif11 or Kif18A was stained using anti-rabbit Kif11 or anti-rabbit Kif18A antibodies, respectively (red). Mouse anti-α-tubulin antibody was used to stain α-tubulin (green), and cell nuclei were visualized using Hoechst (blue). Images represent single medial slices.

Given the previous implication of Kif11 involvement, along with other mitotic kinesins, such as Kif3a, Kif18a, Kif23, and Kif25, in HPV infection (27, 34), we treated HaCaT cells infected with EdU-labeled WT PsV16s for 24 h with the Kif11 inhibitor dimethylensastron (Eg5 inh+) (56) for 10 min. We acquired high-resolution images of untreated (“Ctrl”) and Kif11 inhibitor (“Eg5 inh+”)-treated cells after immunofluorescence staining to detect EdU-labeled pseudogenomes (red) on astral and spindle microtubules (white) in proximity to Kif11 protein (green) during prophase or pro-metaphase (Fig. 4A). Additionally, we quantified the number of EdU puncta in whole cells by counting EdU puncta throughout the cells (Fig. 4B), +/−Kif11 inhibitor, and found no significant difference of EdU puncta throughout the cells between either condition (Fig. 4C). However, we observed a modest but significant difference in the percentage of EdU puncta located on mitotic chromosomes on control (Ctrl) versus Kif11 inhibitor-treated (Eg5 inh+) cells, approximately 29% versus 24%, respectively; this corresponds to an approximate 22% decrease of HPV genomes in Eg5 inh+ cells (Fig. 4D). To address if this decrease of EdU puncta seen with Kif11 inhibitor-treated cells was due to nonspecific inhibition of other kinesins implicated as being involved in HPV intracellular trafficking, staining of Kif11 (red) and Kif18A (red) was analyzed on mitotic chromosomes and on microtubules (α-tubulin staining, green) in cells treated with +/−Kif11 inhibitor. There was no detectible Kif11 on MTs in mitotic cells after Eg5 inh+ treatment (Eg5 inh+ panels, Fig. 4E), whereas Kif18 remained associated on MTs regardless of Kif11 inhibitor treatment (Ctrl and Eg5 inh+ panels, Fig. 4E). Taken together, these data suggest that Kif11 kinesin is at least partially needed for the trafficking of HPV pseudogenomes toward mitotic chromosomes.

### Kinesins Kif11, Kif18a, and Kif25 are in close proximity to HPV16 L2 protein during infection

**Fig 5:**
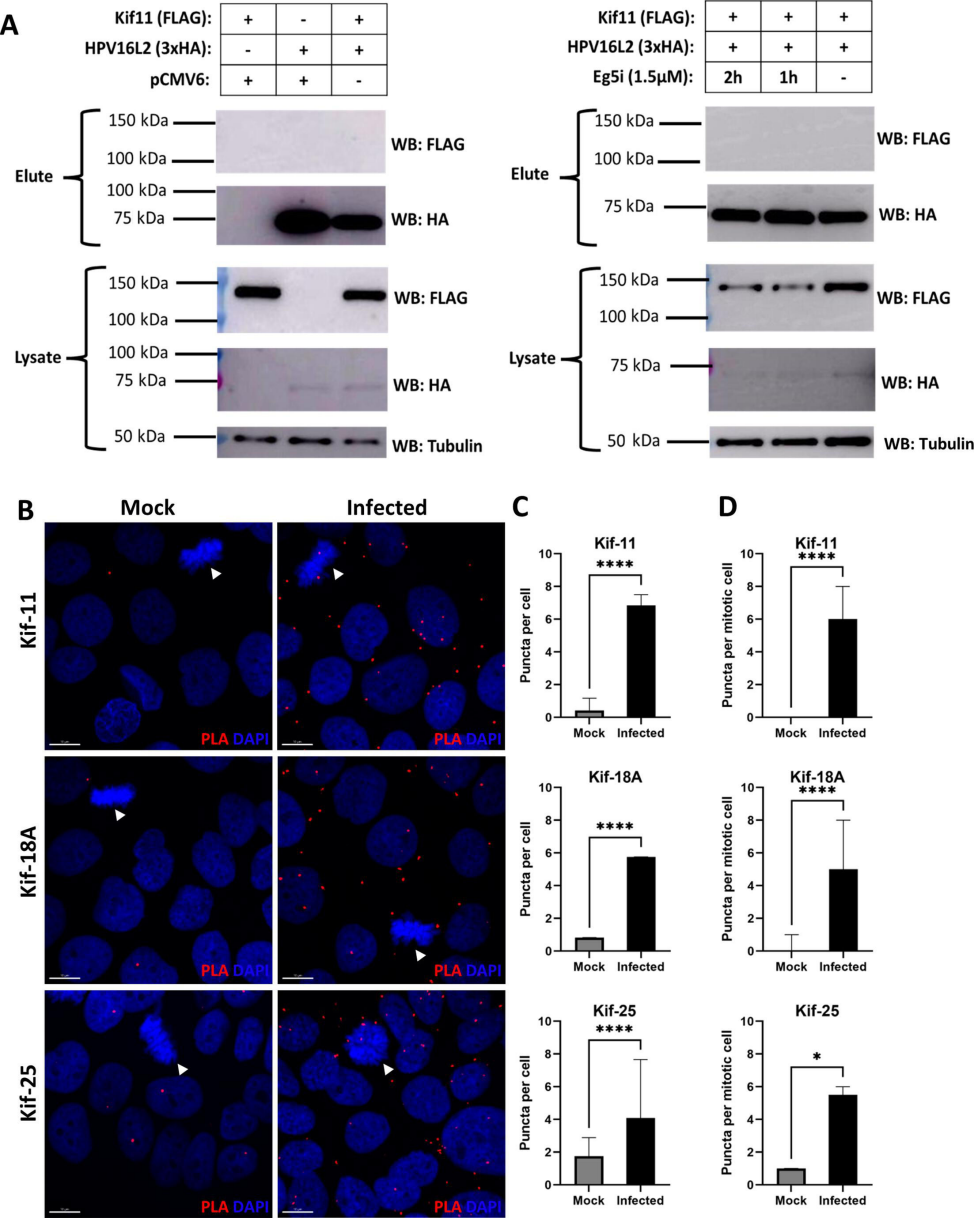
Kinesin motor proteins are in proximity to HPV16 L2 protein during infection. (**A**) HEK293TT cells were transfected with plasmids encoding HA-tagged HPV16 L2 and FLAG-tagged Kif11 to perform a co-immunoprecipitation (CoIP) assay using anti-HA beads; pCMV6 plasmid was used for the CoIP empty vector controls. CoIPs were performed with (right-hand panel) or without (left-hand panel) transfected cells treated with Kif11 inhibitor (1.5 µM; “Eg5i”) 1 or 2 h prior to cell harvesting for CoIP. Tagged HPV16 L2 and Kif11 proteins were probed for via Western blot using anti-HA and anti-FLAG antibodies, respectively, in both whole-cell lysates (“Lysate”) and anti-HA bead elutions (“elute”); anti α-tubulin Western blots were used as a loading control for the lysates. The enclosed immunoblots are representative of at least two independent experiments (*N* = 2). (**B**) Mock-infected (“Mock”) and PsV16-infected (“Infected”) HaCaT cells were processed using the proximity ligation assay (PLA) protocol 21 hpi. PLAs were performed using an antibody cocktail to target the HPV16 L2 protein and an individually selected kinesin, either Kif11, Kif18a, or Kif25. The HPV16L2 antibodies used are the same throughout all PLAs, while the specific kinesin antibody used for the indicated PLA is identified in the left-hand margin of the images. PLA puncta were visualized in the representative images as red dots. Confocal images were acquired as z-stacks and processed as described in Materials and Methods. The white arrows highlight mitotic cells in their representative images for the PLAs. (**C**) The average PLA puncta numbers per interphase cells were counted for at least 200 cells per group in one experiment and are presented as median with 95% CI in the corresponding graphs for the PLAs in the middle panels adjacent to their representative images. The average PLA puncta numbers per mock-infected versus PSV-infected interphase cell were (median) 0.43 vs 6.8 (Kif11), 0.83 vs 5.8 (Kif18A), and 1.8 vs 4.1 (Kif25). Differences between mock and infected cells were analyzed using Mann–Whitney test, (*N* = 3); ****P < 0.0001. (**D**) The average PLA puncta numbers were counted per at least 20 mitotic cells for each condition and are presented as median with 95% CI. Mock-infected vs PSV-infected PLA puncta numbers per mitotic cell were (median) 0.0 vs 6.0 (Kif11), 0.0 vs 5.0 (Kif18A), and 1.0 vs 5.5 (Kif25). Differences between mock and infected cells were analyzed using Mann–Whitney test, ****P < 0.0001.

To further ascertain a direct interaction between Kif11 and HPV16 L2, we performed a co-immunoprecipitation (CoIP) between FLAG-tagged Kif11 and HA-tagged HPV16L2 overexpressed in HEK293TT cells. However, we did not detect tagged Kif11 with HA-tagged HPV16 L2 in L2-mediated CoIPs (Fig. 5A). In lieu of not being able to show a direct interaction between Kif11 and L2 via CoIP, we utilized proximity-ligation assays (PLAs) to determine if Kif11 and HPV16L2 are in close proximity during infection, as used previously in HPV16 entry studies (21, 23, 29). We analyzed PLA puncta (red) in both interphase (Fig. 5B and C) and mitotic (Fig. 5B and D) cells. For Kif11-L2 mediated PLA puncta, we observed a 16-fold increase in HPV16-infected interphase cells (Fig. 5C) and a sixfold increase in HPV16-infected mitotic cells (Fig. 5D) compared with mock-infected cells. We repeated this PLA analysis with kinesins Kif18a and Kif25, since previously published data suggest that HPV requires these kinesins during infection (27, 34). We found a sevenfold and a twofold increase of Kif18a-L2 and Kif25-L2 PLA puncta in HPV16-infected interphase cells, respectively, and an approximate fivefold increase of both Kif18a and Kif25-L2 puncta in HPV16-infected mitotic cells, respectively, compared with mock-infected cells (Fig. 5C and D).

### Relocalization of Kif11 via knocksideways decreases HPV16 genome deposition on mitotic chromatin and cell infectivity

**Fig 6:**
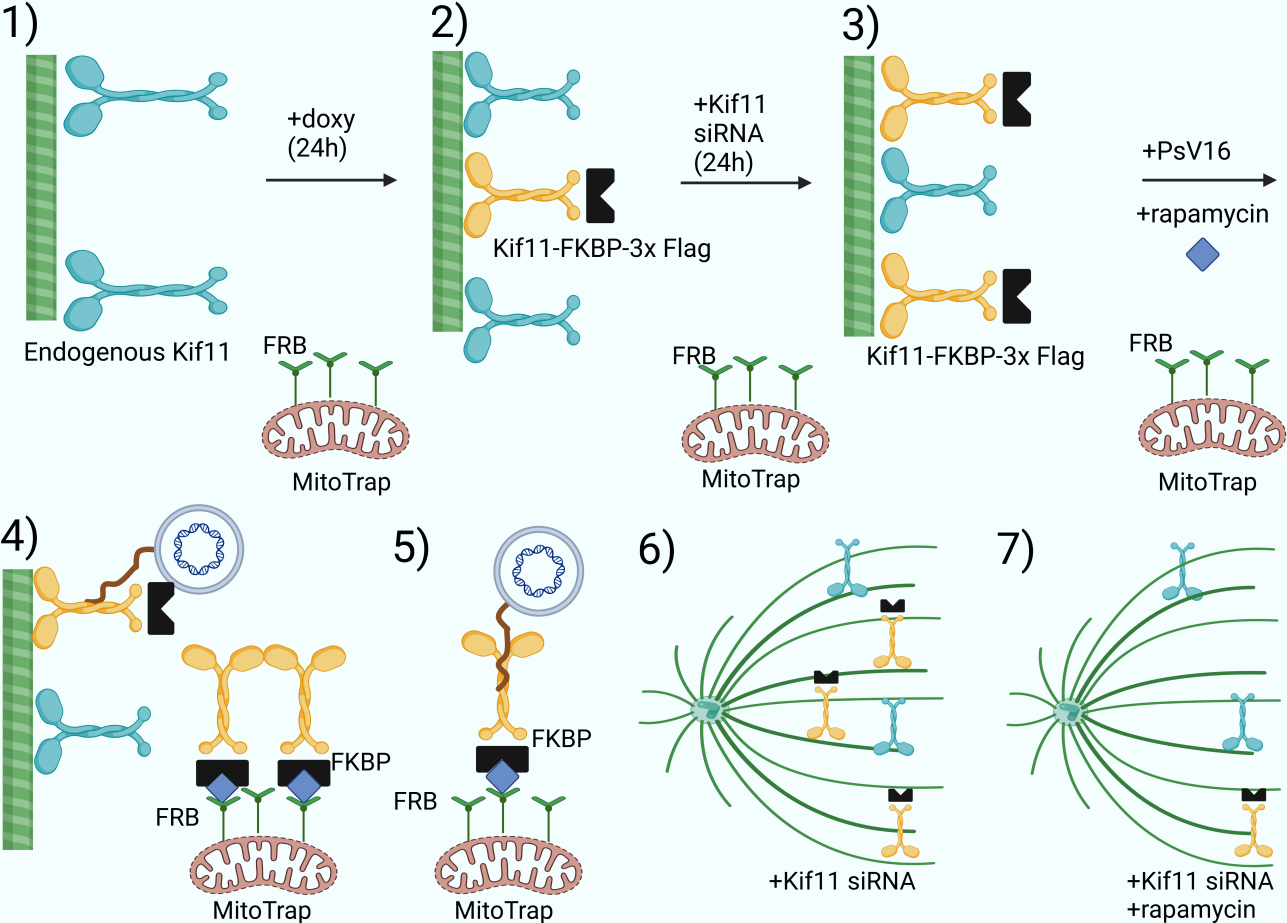
Knocksideways experimental layout. 1) HeLa(Mito)–Kif11–FKBP–3x Flag cells were first treated with doxycycline (doxy) for 24 h to induce expression of the Kif11–FKBP–3x Flag transgene. 2) An endogenous Kif11-targeting siRNA pool was then reverse-transfected into doxy-treated cells to decrease endogenous Kif11 population over 24 h. 3) The doxy and Kif11 siRNA-treated cells, now containing reduced levels of endogenous Ki11 and increased amounts of exogenous Kif11–FKBP–3x Flag protein, were then infected with EdU-labeled PsV16s for either 16 h or infected with PsV16s containing a luciferase reporter genome for 24 h. Infected cells were then treated 16 hpi with 200 nM rapamycin for 1 h or added along with PsV16s for the longer 24 h infection. 4) Rapamycin-induced relocalization of Kif11–FKBP–3x Flag proteins to MitoTrap via dimerization of their respective FKBP and FRB domains. Not all Kif11–FKBP–3x will be re-localized to MitoTrap, some transgenic Kif11 protein will remain on microtubules to transport HPV16. 5) Optimally, the initial goal of the knocksideways experiment was to detect relocalization of EdU-labeled HPV16 genome to mitochondria in infected HeLa(Mito)–Kif11–FKBP–3x Flag cells treated with doxy and rapamycin. 6) Without rapamycin, Kif11–FKBP–3x Flag should perform the same functions as endogenous Kif11, especially during mitosis. 7) With rapamycin addition, a significant portion of the Kif11–FKBP–3x Flag cellular pool will be relocalized to MitoTrap. As a result of cells containing less endogenous Kif11, due to Kif11 siRNA, rapamycin treatment would increase levels of monoastral cells due to less endogenous and transgenic Kif11 available to assist in spindle microtubule sliding. Diagram was generated using BioRender and its available templates.

Given our data showing that HPV16 L2 is in proximity to Kif11 in interphase and mitotic cells (Fig. 5B through D), but yet unable to discern if these two proteins form a complex (Fig. 5A), we co-opted the knocksideways protocol from the work of Robinson et al*.* (57, 58) as an orthogonal approach to directly link Kif11 with plus-end trafficking of the HPV16 genome. The overall layout of this approach is to replace endogenous Kif11 with exogenous Kif11, which is tagged with FK506 binding protein (FKBP) and FLAG (final product: Kif11–FKBP–3x FLAG). Our adaptation of the knocksideways approach and subsequent experimental layout is depicted in Fig. 6. Briefly, we procured HeLa cells stably expressing MitoTrap protein, HeLa(Mito) cells, which were generated as described in Motley et al*.* (59). MitoTrap is a fusion protein containing a mitochondrial-targeting domain (Tom70p) along with an FKBP12 and Rapamycin Binding Protein (FRB) domain. With rapamycin treatment, FKBP fusion proteins relocalize to the mitochondria.

Under our knocksideways setup, HeLa(Mito) cells were stably transduced with a construct encoding the exogenous Kif11–FKBP–3x Flag fusion protein, which is also under control of a doxycycline-inducible promoter (Tet-ON system). We then induced expression of exogenous Kif11 by treating our new HeLa(Mito): Kif11–FKBP–3x Flag cells with doxycycline for 24 h, followed by siRNA-mediated knockdown of endogenous Kif11 for 24 h. The exogenous Kif11–FKBP–3xFlag construct was designed to be resistant to Kif11-targeting siRNAs. Cells were then infected with EdU-labeled PsV16s for 16 h. After 16 hpi, infected cells were treated with rapamycin for 1 h to relocalize the exogenous Kif11 fusion protein to MitoTrap via rapamycin-induced dimerization of FRB-containing MitoTrap with FKBP-containing Kif11. These cells were then processed for microscopic analysis. Mitotic cells were then examined for HPV16 pseudogenome deposition by measuring EdU puncta. In a parallel experiment, rapamycin was added along with infection using luciferase reporter-containing PsV16s for 24 h, and PsV16 infectivity was then determined via luciferase reporter activity assays.

**Fig 7:**
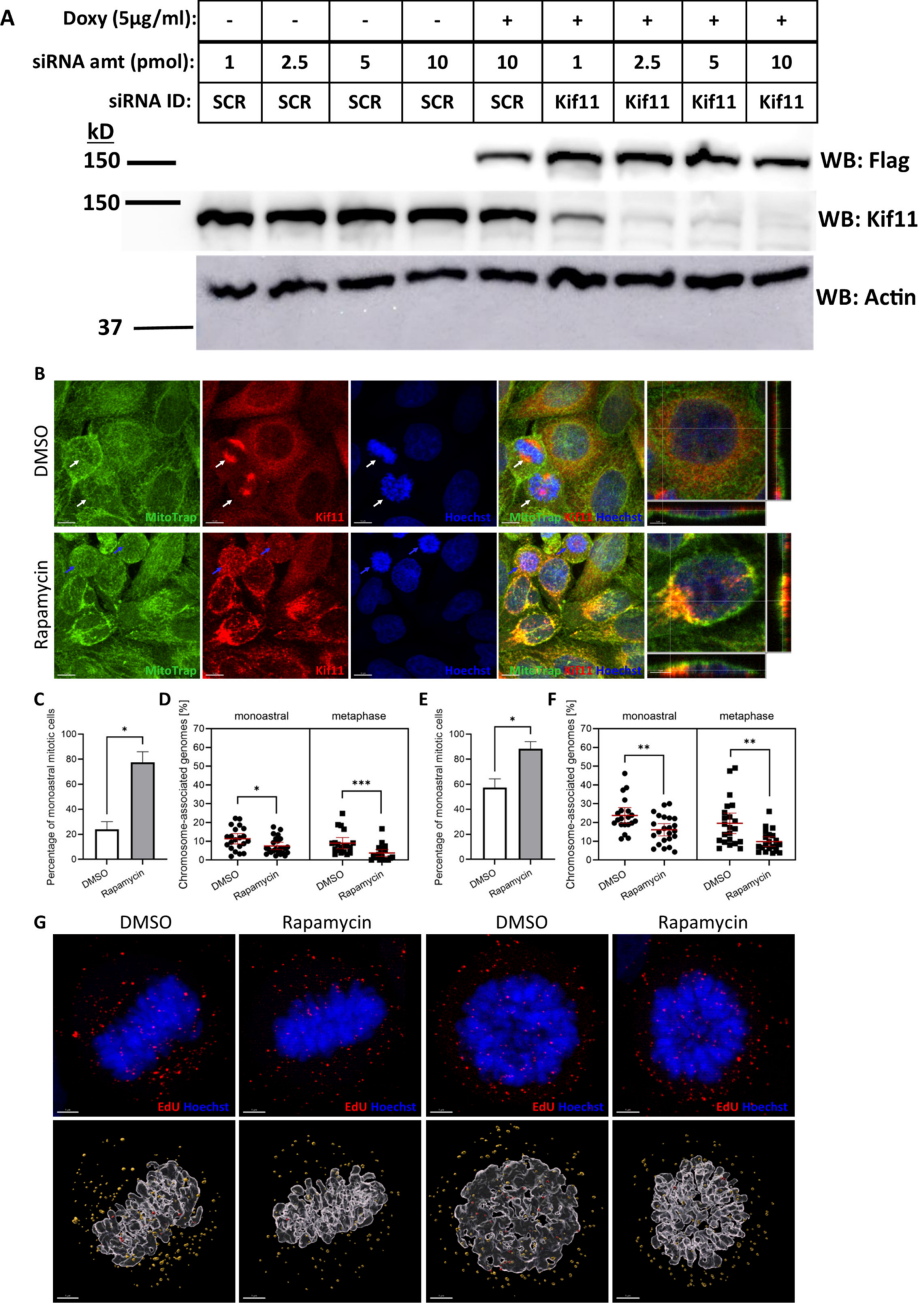
Relocalization of Kif11 via knocksideways significantly decreases transport of EdU-labeled PsVs to mitotic chromosomes. HeLa(Mito) cells containing the tet-inducible Kif11–FKBP–3x Flag construct, were treated with doxycycline (doxy, 5 µg/mL) for 24 h, reverse-transfected with 2.5 pmol of Kif11-targeting siRNA for 24 h, and then infected with PsV16 for 16 h. Cells were then treated with either DMSO or rapamycin (200 nM) for 1 h prior to processing for confocal microscopy or Western blotting (WB) as described in Materials and Methods. (**A**) Doxy-treated (24 h) HeLa(Mito) cells were harvested approximately 42 h post-siRNA-transfection (1–10 pmol control scrambled (SCR) or Kif11-targeting [Kif11] siRNAs) and probed via WB for presence of endogenous Kif11 (WB:Kif11) or exogenous Kif11–FKBP–3x Flag (WB: Flag). Blot is representative of two separate experiments. (**B**) For panels left to right, for HeLa(Mito): Kif11–FKBP–3x flag cells treated with doxy, than 2.5 pmol Kif11 siRNAs, and finally treated with either DMSO or rapamycin; mouse anti-HA antibody was used to stain for MitoTrap protein (green), rabbit anti-Kif11 was used to stain for Kif11 (red), and nuclei were stained with Hoechst (blue). Images with combined staining are on the right-hand side, with a close-up of a selected cell. Colocalization of Kif11 and MitoTrap are visualized as yellow puncta in the combined image, with representative signal(s) shown in the z-stacks provided with the close-up images. White arrows in the DMSO panels show metaphase cells, and blue arrows in the rapamycin panels indicate monoastral cells. (**C,E**) The percentages of monoastral cells for each condition (DMSO or rapamycin treatment) were determined by Hoechst33342 staining. Confocal images were acquired as z-stacks with Olympus CSU W1 Spinning Disk Confocal System using a 20× objective. Monoastral quantifications in (**C**) and (**E**) are based on Hoechst staining and represent individual experiments. Differences in monoastral cell percentages in each condition (DMSO or rapamycin treatment) were analyzed using the Mann–Whitney test; *, *P* < 0.05. (**D, F, G**) Quantification of EdU-labeled PsV16(s) on mitotic chromatin of either monoastral or metaphase cells, with or without rapamycin treatment. Approximately 23 cells per condition per experiment were counted, with (**D**) 11, 6.3, 7.4 , and 2.2 (median) EdU puncta counted in monoastral, −/+ rapamycin and metaphase, −/+ rapamycin-treated cells, respectively, in one independent experiment and (**F**) 22, 16, 17, and 8.0 (median) EdU puncta counted in monoastral, −/+ rapamycin and metaphase, −/+ rapamycin-treated cells, respectively, in another independent experiment. (**G**) Confocal images were acquired and processed as described in Materials and Methods. AF555 dye coupled with Click-iT® chemistry was used to stain EdU-labeled pseudogenomes (red), and genome localization was determined by proximity to mitotic chromatin stained with Hoechst (blue). Differences of EdU puncta amounts on mitotic chromosomes in each condition were analyzed using the Mann–Whitney test; *, *P* < 0.05; **, *P* < 0.01; ***, *P* < 0.001.

Western blotting confirmed expression of the Kif11–FKBP–3x FLAG transgene upon doxycycline (“Doxy”) supplementation. Transfection with Kif11-targeting siRNAs dramatically decreased endogenous Kif11 protein levels, but not the levels of the exogenous fusion protein (Fig. 7A). Rapamycin treatment resulted in relocalization of exogenous Kif11 to Mitotrap protein (Fig. 7B). As expected, rapamycin-dependent relocalization of Kif11 caused approximately 80% of the cells in two independent experiments to enter the monoastral phenotype. We also noted that a significant percentage of cells (20% and 55% per experiment) were monoastral without rapamycin treatment (Fig. 7C and E), suggesting that either the fusion protein cannot fully compensate for endogenous Kif11 or not all cells express the exogenous Kif11. Next, we quantified the number of EdU puncta on mitotic chromosomes in both monoastral cells and cells in metaphase, under DMSO or rapamycin treatment, in the same way as in Fig. 4B and D. Significantly less EdU puncta were seen on mitotic chromosomes in both monoastral and metaphase cells after rapamycin treatment vs DMSO treatment in both independent experiments (Fig. 7D and F). In none of our careful confocal analysis of infected HeLa(Mito) cells subjected to the knocksideways protocol did we observe obvious EdU puncta colocalizing with MitoTrap upon rapamycin treatment (Fig. 7B and G).

**Fig 8:**
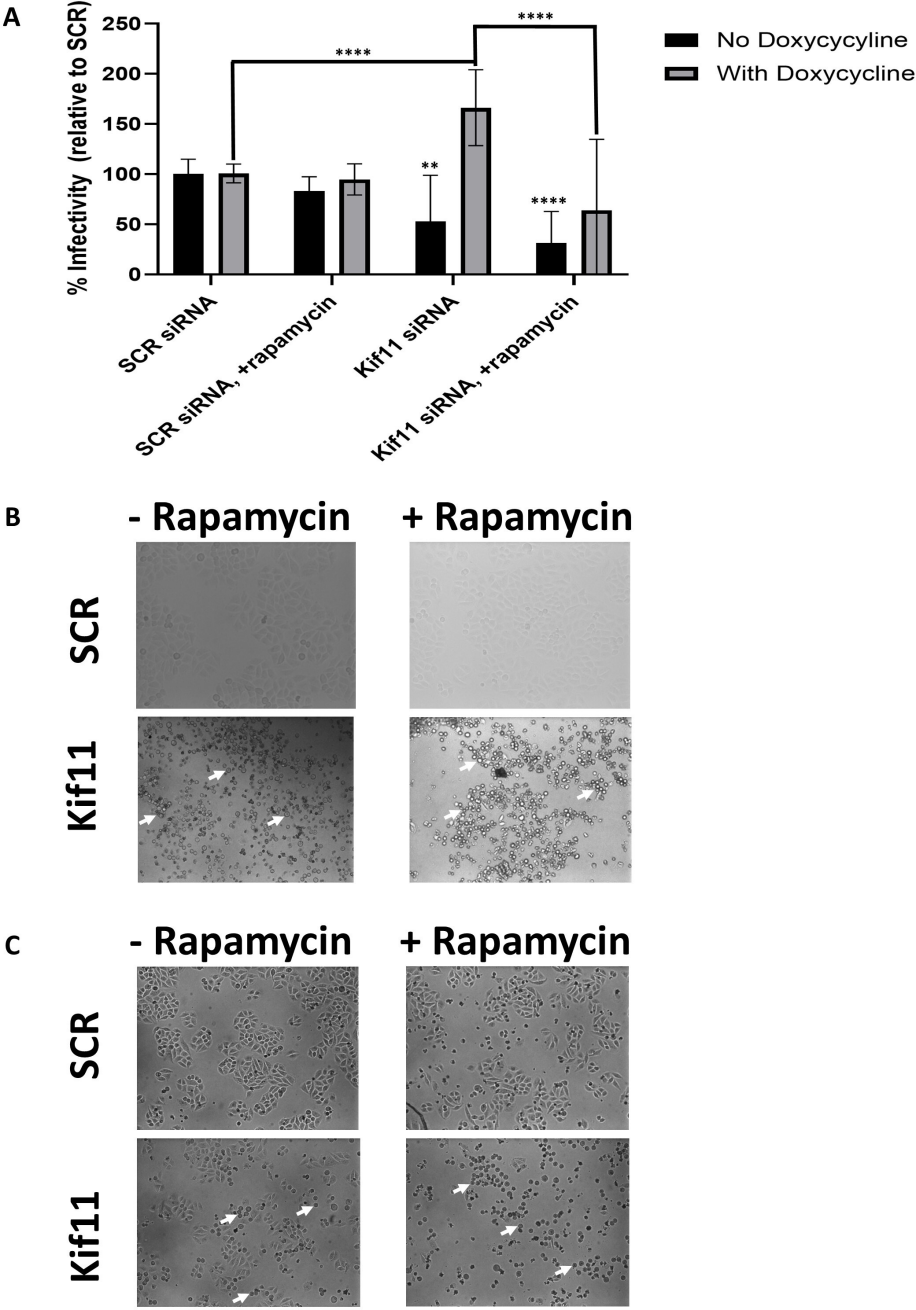
Knocksideways relocalization of Kif11 over 24 h also significantly decreases PsV infectivity of cells. HeLa(Mito): Kif11–FKBP–3x Flag cells were subjected to the same general knocksideways protocol as in Fig. 7, with the main differences being the infection time, and rapamycin time treatments were extended, 24 h each; the resultant infection readout is based on luciferase activity instead of EdU signal on mitotic chromosomes. (**A**) HeLa(Mito) cells containing the tet-inducible Kif11–FKBP–3x Flag construct were treated with or without doxycycline (doxy, 5 µg/mL) and then transfected with control (SCR) or Kif11-targeting siRNAs as before. Cells were then infected with luciferase-containing PsV16s at a VGE of 500 with or without rapamycin (200 nM). At 24 hpi, cells were harvested and lysed using OneGlo substrate, with subsequent luciferase activity ascertained with a Tecan Spark plate reader. “100%” infectivity was set for the luciferase activity of infected cells transfected with control (SCR) siRNAs, with or without doxycycline; the luciferase activity (infectivity) of infected cells for subsequent conditions, with or without doxycycline, was charted relative to the respective “100%” infectivity set points of SCR siRNAs. The % infectivity and standard deviation values for SCR siRNA transfection, −/+ rapamycin, and Kif11siRNA transfection, −/+ rapamycin, under “no doxycycline” conditions were 100.0% ± 14.8, 82.9% ± 14.5, 52.7% ± 46.2, and 31.5% ± 31.3, respectively. The % infectivity and standard deviation values for SCR siRNA transfection, −/+ rapamycin, and Kif11siRNA transfection, −/+ rapamycin, under “with doxycycline” conditions were 100.7% ± 9.3, 94.8% ± 15.5, 166.2% ± 37.9, and 64.1% ± 70.5, respectively. Differences of “% Infectivity” amounts in each condition were analyzed using Student’s *t*-test (*N* = 4, with technical triplicates per condition); **, *P* < 0.01; ****, *P* < 0.0001. (B and C) Representative images of cells (10× magnification) in the stated conditions (−/+ rapamycin, with SCR or Kif11-siRNA transfected cells) in the (**B**) “no doxycycline” or (**C**) “with doxycycline” conditions were taken immediately before cell harvesting and luciferase activity determination. The white arrows show representative cells and clusters of cells that are in the monoastral phenotype. Brightfield images were acquired with a Leica DMI6000 B microsope using a 10x objective.

Next, we followed up by performing a longer knocksideways protocol, *i.e.,* 24 h of infection with PsV16s containing luciferase reporter genome, either with or no rapamycin treatment of the infected cells over the infection time. While rapamycin treatment alone did not significantly decrease PsV16 infectivity of cells, knockdown of Kif11 with or without rapamycin in Kif11–FKBP–3x Flag uninduced cells significantly decreased cell infectivity to 52% and 31%, respectively, as compared with the “no doxycycline” SCR control cells (Fig. 8A). However, induction of exogenous Kif11–FKBP–3x Flag, “with doxycycline” conditions, rescued the infectivity of cells whose endogenous Kif11 was targeted via siRNAs to 166% infectivity as compared with the “with doxycycline” SCR control. This infectivity rescue was significantly ablated with rapamycin treatment to 64.1% infectivity as compared with the “with doxycycline” SCR control (Fig. 8A). Given the number of doxycycline-treated cells in the monoastral phenotype we previously detected without rapamycin treatment (Fig. 7C and E), we took 10× images of infected cells without or with doxycycline treatment, (Fig. 8B and C, respectively), with or without rapamycin treatment, for comparisons prior to luciferase activity determination. Cells whose endogenous Kif11 was knocked-down without exogenous Kif11–FKBP–3x Flag induction had very high levels of monoastral cells, with or without additional rapamycin treatment (Fig. 8B). In contrast, cells treated with doxycycline and Kif11-targeting siRNAs had some monoastral cells, but most of the cells appeared attached; the addition of rapamycin caused most of these doxycycline-treated cells to go into the monoastral phenotype (Fig. 8C).

## DISCUSSION

Our study sought to further elaborate the roles MTs, dynein, and kinesins play on HPV trafficking. While snap-shot analyses have shown that HPV genomes localize near MTs during mitosis, indicating MT-mediated transport, the exact mechanism(s) and the role of dynein and kinesins in HPV genome transport have not yet been fully elucidated (35, 50). This is partially due to vesicular trafficking requiring MT formation prior to mitosis initiation for transport from the TGN, making knockout approaches or long-term inhibitor exposure problematic. We addressed this limitation by treating HPV-infected cells short-term with specific inhibitors against dynein and a specific kinesin, focusing our quantitative analysis on mitotic cells. We provide high-resolution microscopy clearly showing EdU-labeled HPV genomes on astral and spindle MTs, along with more microscopic evidence showing that dynein is required for minus-end directed trafficking to the MTOC in prophase. We investigated the involvement of Kif11 in the trafficking of HPV pseudogenomes at later stages of mitosis due to Kif11 being implicated in HPV infection via RNAi screen (34) and having a readily available inhibitor for Kif11. We also utilized PsVs harboring a previously identified mutation in the L2 protein, R302/5A, in our studies because these PsVs are known to be impaired in trafficking along spindle MTs (28, 35, 54). Short-term treatment of PsV-infected HaCaT cells with the Kif11 inhibitor dimethylenastron significantly decreased the number of HPV pseudogenomes on mitotic chromatin, suggesting the Kif11 kinesin is partially required for plus-end trafficking towards the mitotic chromosomes. We next utilized PLA to determine that several mitotic kinesins, including Kif11, are in close proximity with the L2 protein of HPV16 during infection in both interphase and mitotic cells. Finally, we appropriated a knocksideways approach in a novel attempt to demonstrate a direct interaction between HPV16 L2 and Kif11. While we were not able to use knocksideways to show a direct interaction between L2 and Kif11 via relocalization of HPV genomes to mitochondria, we were able to demonstrate that relocalization of exogenous Kif11 decreased the number of viral puncta on mitotic chromosomes and general infectivity of cells undergoing mitosis and cells trapped in a monoastral phenotype.

None of the data herein contradict the most recent model developed by Lai et al*.* identifying the cellular factors Ran-binding protein 10 (RanBP10) and karyopherin alpha2 (KPNA2) complexing with L2 to facilitate minus-end transport toward the MTOC via dynein (53). Our main novel finding here implicates Kif11 in HPV plus-end transport toward the mitotic chromatin. We initially hypothesized Kif11 as the main kinesin involved in HPV plus-end movement, but the following data we collected forced us to re-evaluate our initial hypothesis: the significant, yet not near-complete disappearance of EdU puncta on mitotic chromosomes upon either inhibitor-mediated dissociation or knocksideways-induced relocalization, respectively, of Kif11 from MTs (Fig. 4D, 7D, and 7F,), our inability to CoIP Kif11 and L2 (Fig. 5A), and that we have never detected relocalization of HPV genome to MitoTrap protein with our knocksideways approach as described in Fig. 7.

We propose two models for the involvement of Kif11 and other kinesin(s), such as Kif18s and Kif25, in HPV genome trafficking. One model imputes a role for these kinesins in the stabilization of HPV genome transport along MTs, given that trafficking on MTs is complex. While overall motion is polarized, cargo on MTs has been observed to stop and change direction(s) back and forth in a zig-zag pattern. Cooperative movement of multiple kinesins transporting cargo(es) seems to allow for smoother transport (41, 60). Kinesin stabilization of HPV genome transport could be important in both interphase and mitotic cells, thus explaining our PLA data in both cell groups (Fig. 5B through D). For the other model, we propose that Kif11 acts as a “switching station” whereby L2 briefly interacts with Kif11 recruited to and enriched on the spindle microtubules, near MTOCs during mitosis, allowing the HPV genome-containing vesicles to properly orient themselves onto the spindle microtubules. Other mitotic kinesins, including Kif18a and Kif25, then complete the plus-end HPV transport to the mitotic chromosomes. Perturbation of Kif11 either via inhibitor or the knocksideways protocol significantly decreased HPV16 pseudogenome deposition on mitotic chromatin or general cell infectivity (Fig. 4D, 7D, 7F and 8A). However, these results do not conclusively prove that this phenotype is solely due to the loss of Kif11 interfering with HPV transport along MTs near MTOCs, as if Kif11 is involved in any trafficking step prior to genome association with chromatin, one would see the same decrease in infectivity or HPV genome deposition upon Kif11 disruption. The roughly 20% decrease of HPV16 pseudogenome accumulation on mitotic chromatin with short-term Kif11 inhibition (Fig. 4D) or short-term knocksideways-induced Kif11 relocalization (Fig. 7D and F), along with the finding that Kif11 is in proximity to L2 in infected interphase cells (Fig. 5C), might indicate that Kif11 and other kinesins are important for stabilization. Also, the PLA data in mitotic cells (Fig. 5D) may indicate that L2 is in proximity to other kinesins, such as Kif18a and Kif25, that ultimately have nothing to do with HPV genome trafficking.

Our proposed HPV trafficking models not only fit the enclosed data, but also neither clash with the known biochemical properties of Kif11 or its role in mitosis. Kif11 levels are thought to reach their peak during mitosis and Kif11 itself preferentially binds spindle microtubules near MTOCs (61–63). This localization of Kif11 on spindle MTs during prophase gives L2 a prime platform for our proposed directionality switch. After Kif11 accumulation near the MTOCs, it then forms homotetramers to crosslink MTs and generates force(s) to separate the spindle poles. Given the homotetramerization of Kif11 and that Kif11 localizes at different locations along MTs as mitosis progresses (MOTCs at metaphase, central spindle at anaphase, midbody at anaphase) (64–67), it cannot be the main transporter of the HPV genome to the mitotic chromosomes.

More studies will be required to determine the precise role of Kif11 kinesin and other kinesins, including potential adaptor proteins, involved in plus-end trafficking of HPV genomes to the mitotic chromosomes; further elucidation of post-TGN HPV trafficking to the MTOC via minus-end movement along MTs will also be needed. Since mitosis is absolutely required and a rate-limiting step in HPV entry (33, 34), this limits the types of experimental approaches we can perform to investigate HPV trafficking. Our inhibitor studies have inherent limitations particularly in that there is a lack of specific dynein and kinesin inhibitors (68–70). Despite the short duration of inhibitor treatments and focusing our analysis on a subset of cells undergoing mitosis, we were still able to observe a significant 22% decrease of EdU-labeled HPV pseudogenomes on mitotic chromatin in infected cells after a short incubation with dimethylenastron (Fig. 4D). We also note a significant decrease of EdU-labeled HPV pseudogenome minus-end movement after a short incubation with ciliobrevin D (Fig. 3B). In contrast, Lai et al*.* performed longer-term treatment (at least 1 h) of HPV16-infected HeLa cells with ciliobrevin D at a higher concentration (100 µM) to decrease the relative infection of PsV16s by only roughly 50% (53).

Future studies should also focus on finding alternative ways to identify more kinesins and associated adaptor proteins involved in HPV trafficking, given the listed inhibitor study limitations. We have shown unexpected success with our Kif11 knocksideways approach. While either 20% or 55% of our cells under Kif11 knocksideways conditions went into a monoastral phenotype without rapamycin treatment (Fig. 7C, 7E and 8C), we have demonstrated that FKBP-tagged Kif11 can be successfully relocalized to MitoTrap, and that this relocalization has a dramatic effect upon viral genome deposition onto mitotic chromosomes. To our knowledge, this is the first example of a knocksideways approach being used on a kinesin and in service to a virology study. The large percentages of monoastral cells detected with induced exogenous Kif11, but without rapamycin, indicates that either Kif11–FKBP–3x Flag protein cannot completely compensate for endogenous Kif11, or not all cells expressed exogenous Kif11. Still, our success with the Kif11 knocksideways setup show that other kinesins, such as Kif18a and Kif25, could be substituted for Kif11. The possible limitations here are that there are about 45 known kinesins encoded by the human genome (70, 71) and that knockdown of endogenous kinesin(s) may lead to a compensatory effect by other kinesins due to redundant pathways, confounding results. Lai et al*.* utilized a common method to find novel L2 binding partners by overexpressing L2, identifying L2-interacting proteins via CoIP and mass spectrometry, and then confirming top candidates, such as RanBP10, in HPV trafficking via confocal microscopy coupled with knockdown approaches. Lai et al*.* identified a kinesin-binding partner to the L2 protein, Kif20B; however, Kif20B knockdown did not inhibit HPV pseudogenome trafficking into the nucleus (53). Also, individual kinesin(s) binding to L2 during trafficking might not bind with a strong enough affinity to allow for their isolation by CoIP and subsequent identification by mass spectrometry.

While we employed PLA to determine Kif11, Kif18a, and Kif25 are in proximity to L2 during infection (Fig. 5B through D), PLA experiments depend on the availability of good quality antibodies (72) and prodigious amounts of pseudovirus. It would also be difficult to use PLA to determine if multiple kinesins or other proteins are in proximity with L2 at the same time. However, it may be possible to expand this proximity concept to find additional interacting kinesins in an unbiased manner by tagging L2 with a proximity labeling tag such as BioID2 (73, 74). BioID2 has recently been utilized to map out a proximity network between proteins involved in the spindle assembly checkpoint (75). Proximity-labeling could also identify adaptor proteins used by L2-adjacent molecular motors, as they could potentially affect HPV trafficking on dynein and kinesin(s); HIV utilizes the kinesin-adaptor protein FEZ1 to effectuate kinesin 1-mediated trafficking into the nucleus (49).

In summary, we have presented data that further elucidate the mechanism used by HPV-containing vesicles to traffic along MTs to the nucleus. We confirmed previously published data that dynein is required for minus-end-directed transport from the TGN to the MTOC during prophase (53). We demonstrate partial colocalization between the kinesin Kif11 and HPV genome from PsVs containing WT HPV16 L2. Next, we show Kif11 inhibitor, PLA, and novel Kif11 knocksideways data that support two potential models explaining how Kif11 effectuates HPV genome trafficking, either by trafficking stabilization or by helping correctly position HPV-containing transport vesicles onto spindle microtubules via L2.

## MATERIALS AND METHODS

### Cell lines

The 293TT cells used for the generation of pseudovirions were a kind gift from Dr. John Schiller (Laboratory of Cellular Oncology, National Cancer Institute, Bethesda, MD). 293TT cells were cultured in Dulbecco’s Modified Eagle Medium (DMEM) supplemented with 10% fetal bovine serum (FBS), non-essential amino acids, L-Glutamine, and antibiotic/antimycotic. The immortalized human keratinocytes, HaCaT cells, used for the infection studies were purchased from the American Type Culture Collection and grown in low glucose DMEM containing 5% FBS and antibiotics. HeLa-Mitotrap (Mito) cells were purchased from Sigma-Aldrich (#15042201) and grown in same conditions as the 293TT cells. Media, nonessential amino acids, L-Glutamine, and antibiotic/antimycotic were purchased from Invitrogen (Gibco) and FBS was purchased from Atlanta Biologicals.

### Generation of HPV16 pseudovirions

HPV16 pseudoviruses (PsVs) encapsulating a green fluorescent protein (GFP) expression plasmid (pFWB) or luciferase (pGL3; Promega) were generated in 293TT cells as previously described using pShell16L1L2HA-3′ expression plasmid (76–79). The pFWB plasmid was also kindly provided by Dr. John Schiller. The codon-optimized L1 and L2 expression plasmids were graciously provided by Dr. Martin Müller (Deutsches Krebsfoschungszentrum, Heidelberg, Germany) (80). For pseudogenome detection by immunofluorescent microscopy, pseudogenomes were labeled by supplemented the growth media with 100 µM 5-ethylnyl-2’-deoxyuridine (EdU) at 6 h post-transfection during PsV generation as previously described (81). PsV DNA was isolated using the NucleoSpin Blood QuickPure kit (Macherey-Nagel; #740569.250), supplemented by pre-treating PsVs with 4 µM EDTA and DTT prior to PsV DNA isolation. Genome copy number of PsVs was quantified by quantitative PCR (qPCR). PsVs carrying the R302/5A L2 mutation were generated as previously described (28).

### Antibodies and other reagents

Primary antibodies used for the immunofluorescence studies were as follows: mouse monoclonal antibody (mAb) anti-α-tubulin (Cell Signaling; #3873S), mouse mAb AlexaFluor (AF) 488-conjugated anti-α-tubulin (Cell Signaling; #8058), mouse mAb anti-γ-tubulin for the detection of the MTOC (Sigma-Aldrich; #T6557), rabbit mAb anti-dynein intermediate chain 1 (Abcam; #ab171964); all rabbit mAbs for detection of kinesins such as Kif11 (Cell Signaling; #14404S), Kif18a (Novus Biologicals; #NBP1-85126), and Kif25 (Novus Biologicals; #NBP1-92055). The secondary antibodies used were goat AF-labeled polyclonal Abs (Life Technologies; #A11034, #A21236). Click-iT EdU Imaging Kit (Molecular Probes; #C10338) was used for Click-iT reactions to detect EdU-labeled pseudogenomes. The primary antibodies used to probe for FLAG-tagged and HA-tagged proteins via Western blot were mouse anti-FLAG (Sigma; F-3165) and anti-HA (Sigma; #H9658) antibodies, respectively; the secondary antibody used for Western blots was peroxidase AffiniPure goat anti-mouse IgG (H + L) (Jackson ImmunoResearch Laboratories; 115–035-003). Ciliobrevin D (Calbiochem; #250401) was used to inhibit cytoplasmic dynein by treating cells for 10 min with 50 µM. Kif11 inhibitor III, dimethylenastron (Calbiochem; #324622) was used to inhibit Kif11 by treating cells for 10 min with 1.5 µM of inhibitor. Secondary PLUS/MINUS antibodies used in the proximity ligation assays were provided by the Duolink *In Situ* PLA Probe Anti-Mouse MINUS kit (Sigma-Aldrich; DUO92004) and the Duolink *In Situ* PLA Probe Anti-Rabbit PLUS kit (Sigma-Aldrich; DUO92002). Ligation and amplification reagents also used in the proximity ligation assays were provided by the Duolink *In Situ* Detection Reagents Red kit (Sigma-Aldrich; DUO92008). For lentiviral generation plasmids, psPAX2 was a gift from Didier Trono (Addgene plasmid # 12260; https://www.addgene.org/12260/; RRID:Addgene_12260) and pMD2.G was a gift from Didier Trono (Addgene plasmid # 12259; https://www.addgene.org/12259/; RRID:Addgene_12259).

### Immunofluorescent staining and microscopy

HaCaT cells were grown on glass coverslips at approximately 50% confluency, then infected with EdU-labeled PsVs at approximately 3 × 10^3^ viral genome equivalents (VGE) per coverslip for 24 h at 37°C. Briefly, cells were fixed with 4% paraformaldehyde (PFA) for 15 min at room temperature and then washed with phosphate-buffered saline (PBS; pH 7.5). Next, cells were permeabilized with 0.5% Triton X-100 (TX-100) in PBS for 5 min at room temperature and washed again with PBS. The blocking step for our staining protocol entails adding 5% normal goat serum (NGS) to cells for 15 min at room temperature. To specifically visualize EdU-labeled pseudogenomes, cells were next treated with the Click-iT reaction containing AF555 for 30 min at room temperature protected from light (81). Prior to antibody addition(s), cells were washed again with PBS; incubations with primary antibodies were performed by adding antibodies diluted in 2.5% NGS to cells for 30 min at 37°C in a humidified chamber. After more washing in PBS, cells were incubated with AF-labeled secondary antibodies in 2.5% NGS for 30 min at 37°C in a humidified chamber. Cells were extensively washed again in PBS and then were either mounted in ProLong Gold antifade reagent with DAPI (4′,6-diamidino-2-phenylindole, Invitrogen; #P36931) or first stained with Hoechst 33342 and then mounted with ProLong Glass Antifade Mountant (Invitrogen; #P36984). Confocal images were acquired with a Leica TCS SP5 Confocal Microscope using 63× objective. High-resolution images were acquired with Nikon N-SIM E Super Resolution microscope using 100× objective. Several z-stacks spanning the whole nucleus or cell were acquired and assembled using three-dimensional reconstruction in NIS Elements software. Percent of EdU signal association with target protein(s) or mitotic chromosomes was ascertained by manually counting total number of EdU (red) puncta in the cell over number of EdU puncta partially colocalizing with target protein(s) or mitotic chromatin (38). All images from individual experiments were acquired under the same laser power and reconstruction settings and uniformly enhanced in Adobe Photoshop. Quantifications were performed with IMARIS 9.7.

### Proximity ligation assay

HaCaT cells were infected for 21 h with PsV16s at an approximate VGE of 2 × 10^4^, then subjected to proximity ligation assay (PLA) to determine if several target kinesins, either Kif11, Kif18a, or Kif25, and HPV16 L2 protein are in proximity (≤40 nm) based upon the procedure described in Lin et al*.* (82) and according to the instructions of the manufacturer enclosed with the Duolink *in situ* PLA Probe and Duolink *in situ* detection kits. Briefly, mock-infected and infected cells were stained as previously described, up to and including the Click-iT reaction step without AF555 addition. Next, cells were incubated with a cocktail of antibodies to HPV16 L2 protein and for an individual kinesin, either Kif11, Kif18, or Kif25. We used a 1:800 dilution for the individual kinesin antibodies, a 1:800 dilution for the 33L2-1 antibody, and a 1:1,000 dilution for the K1 and K4 antibodies for the HPV16 L2 antibody cocktail. The cells were incubated with this cocktail of antibodies for 1 h at 37°C in 1× PBS solution, containing 2.5% NGS and 0.1% TX-100, in a humidity chamber. After thoroughly washing slides with 1× PBS, cells were incubated with Duo-link *in situ* PLA Probe PLUS/MINUS secondary antibodies for 1 h at 37°C in humidity chamber, then washed with 0.01M Tris, 0.15M NaCl, and 0.05% Tween-20 (pH7.4) buffer. Cells were then incubated with ligase and ligase buffer from the Duolink *in situ* detection reagents Red kit for 0.5 h at 37°C in humidity chamber and washed again with 0.01M Tris, 0.15M NaCl, and 0.05% Tween-20 (pH7.4) buffer. Finally, cells were incubated with polymerase and amplification solution from the Duolink *in situ* detection reagents kit for 100 min at 37°C in humidity chamber, washed with 0.2M Tris and 0.1M NaCl (pH7.5) buffer, and mounted with DAPI-containing mounting media. Confocal images were then acquired as z-stacks, at least 12 slices per image, with Olympus CSU W1 Spinning Disk Confocal System using a 60× objective. Images were processed using cellSens Software V4.1 and Imaris 9.8.2 Software.

### Coimmunoprecipitation

One million 293TT cells were transfected with 2 µg of FLAG-tagged Kif11 (Ori-gene; #RC218842) and HA-tagged HPV16 L2 overexpression constructs using Lipofectamine 3000 (Invitrogen; #L3000015) protocol. Transfected cells were harvested and lysed using 1% NP-40 Tris-NaCl (pH 8.0) buffer, with lysates incubated with HA magnetic beads (ThermoFisher; #88836) for 1 h at 4°C. After lysate incubation with beads, beads were washed 5× with 1% NP-40 Tris-NaCl buffer, and proteins were eluted from beads via boiling them at 95°C for 10 min. Proteins in lysates and bead elutes were resolved on 10% SDS-PAGE gels, transferred to nitrocellulose, and probed for presence of FLAG-tag, HA-tag, and alpha-tubulin via Western blot.

### Knocksideways protocol

We adapted the knocksideways protocol from the work of Robinson et al*.* (57, 58). First, we generated a transgene of Kif11, whose gene product is a fusion protein of Kif11 with a FK506-binding protein (FKBP) domain and 3× FLAG tags added to the C-terminus of Kif11, using the services of Integrated DNA Technologies (IDT). Additionally, the Kif11 DNA sequence contains silent mutations to render it insensitive to targeting by the pool of Kif11-targeting siRNAs (Dharmacon; L-003317-00-0005) we used to knockdown endogenous Kif11. We further utilized the services of then e-Zvec (now Polyplus) to insert our Kif11–FKBP–3x Flag transgene into a lentiviral vector where Kif11 transgene expression would be under the control of a doxycycline promoter (Tet-ON System). Next, HeLa cells expressing Mitotrap protein, (Mito), were transduced with our Kif11–FKBP–3x Flag construct via second generation lentiviral system using psPAX2 and pMG2.D plasmids. We then isolated a puromycin-resistant (5 µg/mL) clonal population to use for further experiments; Kif11–FKBP–3x Flag expression was induced in these clonal cells with 5 µg/mL doxycycline. After 24 h post-Kif11–FKBP–3x Flag induction, either 1–10pmol of control siRNA (Dharmacon; D-001810-10-05) or Kif11-targeting siRNA pools were reverse-transfected into (approximately) 80,000 cells using Lipofectamine RNAiMax Reagent (Thermo Fisher Scientific; 13778-075). Post-siRNA treatment cells were infected with HPV16 pseudoviruses containing either EdU-labeled GFP-genomes or unlabeled luciferase genomes for 16 or 24 h at approximate VGEs of either 3 × 10^3^ or 500, respectively. Relocalization of exogenous Kif11–FKBP–3x Flag protein was then induced by addition of 200 nM rapamycin for 1 or 24 h. For microscopic analysis after infection, the cells were fixed with 4% PFA and stained for immunofluorescent analysis as previously described. For cells infected with luciferase-containing pseudoviruses, infectivity was determined by lysing infected cells with the ONE-Glo Luciferase Assay System (Promega) and ascertaining luciferase activity using a Tecan Spark plate reader. Knockdown of endogenous Kif11 and expression of exogenous Kif11 were both confirmed via Western blot (1:1000 dilution of anti-Kif11 and anti-FLAG antibodies). Brightfield images were acquired with a Leica DMI6000 B microsope with 10x objective. Confocal images were then acquired as z-stacks, at least 16 slices per image, with a Leica TCS SP5 Confocal Microscope with 63× objective or Nikon N-SIM E Super Resolution microscope with 60× objective. Images were processed using Imaris 10.1 Software. Colocalization between mitotic chromosomes and EdU signal was determined using Imaris spot/surface or surface/surface analysis; distances equal to or lower than “0” between EdU and mitotic chromosomes were counted as a colocalization. Knocksideways diagram was generated using BioRender and its available templates.

## Data Availability

We confirm that all data supporting the findings of this study and underpinning their conclusions are available within the text of the article or the supplemental material or are available upon request.
